# Physicians’ Rebate Check-Book

**Published:** 1892-06

**Authors:** 


PHYSICIANS' REBATE CHECK-BOOK. By Wm. Jefferson Guernsey, M. D., 4340 Frankford Avenue, Philadelphia, Pa. Price, $1.00 per book of 200 Checks.

This publication consists of a series of checks, four to a page, and bound together into books of two hundred each. When a patient comes into the office and pays his fee for a prescription, one of the checks is torn off and given to him, which represents a value of 25 per cent. of the fee. When he has in this way accumulated four checks he can get a prescription by presenting these four checks, and therefore free of actual cost. Thus he has an incentive always to pay his fees immediately. The principle applies also to visits. If he should owe a bill he can, by saving his checks and returning them when he has accumulated a sufficient number, discharge the indebtedness. The loss the physician sustains in this way is abundantly made up by the encouragement offered to the patient to pay as he goes. W. M. J.

